# Molecular Evolution of the H5 and H7 Highly Pathogenic Avian Influenza Virus Haemagglutinin Cleavage Site Motif

**DOI:** 10.1002/rmv.70012

**Published:** 2024-12-27

**Authors:** Jasmina M. Luczo, Erica Spackman

**Affiliations:** ^1^ Australian Animal Health Laboratory Australian Centre for Disease Preparedness Commonwealth Scientific and Industrial Research Organisation East Geelong Australia; ^2^ United States Department of Agriculture Exotic & Emerging Avian Viral Diseases Research Southeast Poultry Research Laboratory United States National Poultry Research Center Agricultural Research Service Athens Georgia USA

**Keywords:** H5, H7, haemagglutinin, haemagglutinin cleavage site motif, highly pathogenic avian influenza virus

## Abstract

Avian influenza viruses are ubiquitous in the *Anatinae* subfamily of aquatic birds and occasionally spill over to poultry. Infection with low pathogenicity avian influenza viruses generally leads to subclinical or mild clinical disease. In contrast, highly pathogenic avian influenza viruses emerge from low pathogenic forms and can cause severe disease associated with extraordinarily high mortality rates. Here, we describe the natural history of avian influenza virus, with a focus on H5Nx and H7Nx subtypes, and the emergence of highly pathogenic forms; we review the biology of AIV; we examine cleavage of haemagglutinin by host cell enzymes with a particular emphasis on the biochemical properties of the proprotein convertases, and trypsin and trypsin‐like proteases; we describe mechanisms implicated in the functional evolution of the haemagglutinin cleavage site motif that leads to emergence of HPAIVs; and finally, we discuss the diversity of H5 and H7 haemagglutinin cleavage site sequence motifs. It is crucial to understand the molecular attributes that drive the emergence and evolution of HPAIVs with pandemic potential to inform risk assessments and mitigate the threat of HPAIVs to poultry and human populations.

**Abbreviations**
AIV: avian influenza virus
Arg: arginine
Glu: glutamic acid
Gly: glycine
gs/Gd: A/goose/Guangdong/1/1996
HA: haemagglutinin
HACS: haemagglutinin cleavage site
HPAIV: highly pathogenic avian influenza virus
IAV: influenza A virus
Leu: leucine
LPAIV: low pathogenicity avian influenza virus
Lys: lysine
M: matrix
MSPL: mosaic serine protease large‐form
NA: neuraminidase
NP: nucleoprotein
NS: non‐structural
PA: polymerase acidic
PB1: polymerase basic 1
PB2: polymerase basic 2
PC: proprotein convertase
pHACS: polybasic haemagglutinin cleavage site
RdRp: RNA dependent RNA polymerase
vRNPs: viral ribonucleoproteins

## 1 Natural History of Highly Pathogenic Avian Influenza Virus

Avian influenza viruses (AIVs) are ubiquitous in certain species of waterfowl and shorebirds in the orders *Anseriformes* and *Charadriiformes* are thought to be the ecological reservoirs of AIVs [1, 2, 3, 4]. AIVs naturally circulate in waterfowl and shorebirds and generally cause little to no disease, suggesting that migratory wild aquatic birds can act as subclinical carriers for the long‐range dispersal of AIVs [5, 6, 7]. Two pathotypes of AIVs are described based on pathogenicity characteristics in chickens or the molecular composition of the haemagglutinin (HA) cleavage site (HACS) motif [8, 9]. Low pathogenicity avian influenza viruses (LPAIVs) generally cause subclinical or mild clinical disease in avian species [9]. In contrast, infection with highly pathogenic avian influenza viruses (HPAIVs) lead to fulminant disease with high mortality rates in gallinaceous species [8, 9]. To date, HPAIVs have emerged in the H5 and H7 subtypes only.

The earliest recorded description of HPAIV in poultry (then known as fowl plague) that caused severe disease and high flock mortality was in 1878 [10]. Early accounts of fowl plague were commonly confused with fowl cholera, however clinical disease differed [11]. In the early 1900s, the etiological agent of fowl plague was determined to pass through filters with pore sizes too small for bacteria, thus a virus [12, 13]. Early descriptions of fowl plague frequently reported cyanosis of the comb, a common sequela of HPAIV infection in chickens. It was not until 1955 that fowl plague virus was determined to be an influenza A virus (IAV) [14] and subsequent sequencing studies towards the latter half of the 20^th^ century confirmed that these ancestral HPAIVs belonged to the H7 subtype [15, 16, 17]. In 1959, an antigenically distinct HPAIV, an H5, was isolated from an epornitic in Scotland [18]. Since 1959 (to November 2024), there have been 54 HPAIV epornitics (Table 1), resulting in substantial economic losses and the culling of millions of birds. Whilst most epornitics have been geographically confined, this has not been the case for the A/goose/Guangdong/1/1996 (gs/Gd)‐lineage. The gs/Gd‐lineage epizootic is unprecedented in its size and geographical spread, and it has been sustained for 20+ years (reviewed in [86, 87, 88]), ignited by spillover transmission from poultry to wild birds then fuelled by the continued circulation of gs/Gd‐lineage HPAIVs in wild birds [7, 89].

**TABLE 1:** Highly pathogenic avian influenza virus epornitics since 1959.

Year	Country	Subtype	Representative isolate	H5 lineage	Representative isolate HACS motif	References
1959	Scotland	H5N1	A/chicken/Scotland/1959	Eurasian (outgroup)	PQ_RKKR/G	[18]
1961	South Africa	H5N3	A/tern/South Africa/1961	Eurasian (outgroup)	PQ_RETRRQKR/G	[19, 20, 21]
1963	England	H7N3	A/turkey/England/1963	—	PE_TPKRRRR/G	[22]
1966	Canada	H5N9	A/turkey/Ontario/7732/1966	Am‐non‐gs/Gd	PQ_RRKKR/G	[23]
1976	Australia	H7N7	A/chicken/Victoria/1976	—	PE_IPKKREKR/G	[24]
1979	Germany	H7N7	A/chicken/Leipzig/1979	—	PE_IPKKKKR/G	[25]
1979	England	H7N7	A/turkey/England/199/1979	—	PE_IPKKREKR/G	[26]
1983–84	USA	H5N2	A/chicken/Pennsylvania/1370/1983	Am‐non‐gs/Gd	PQ_KKKR/G	[27]
1983	Ireland	H5N8	A/turkey/Ireland/1378/1983	Am‐non‐gs/Gd	PQ_RKRKKR/G	[28]
1985	Australia	H7N7	A/chicken/Victoria/1985	—	PE_IPKKREKR/G	[29]
1991	England	H5N1	A/turkey/England/50–92/1991	EA‐non‐gs/Gd	PQ_RKRKTR/G	[30]
1992	Australia	H7N3	A/chicken/Victoria/9306–04‐0930/1992	—	PE_IPKKKKR/G	[31]
1994	Australia	H7N3	A/chicken/Queensland/1994	—	PE_IPRKRKR/G	[32]
1994–95	Mexico	H5N2	A/chicken/Puebla/8623–607/1994	Am‐non‐gs/Gd	PQ_RKRKTR/G	[33]
1994–95	Pakistan	H7N3	A/chicken/Pakistan/447/1995	—	PE_TPKRKRKR/G	[34]
1996 ‐present	China—now global	H5N1/x	A/goose/Guangdong/1/1996 (H5N1, Clade 0) A/gyrfalcon/Washington/41088–6/2014 (H5N8, 2.3.4.4c) A/turkey/Indiana/22–003707‐003/2022 (H5N1, 2.3.4.4b) A/bovine/Texas/24–008749‐002/2024 (H5N1, 2.3.4.4b)	gs/Gd	PQ_RERRRKKR/G PL_RERRRKR/G Refer to GISAID PL_REKRRKR/G	[35, 36, 37]
1997	Australia	H7N4	A/chicken/New South Wales/1/1997	—	PE_IPRKRKR/G	[38]
1997–98	Italy	H5N2	A/chicken/Italy/330/1997	EA‐non‐gs/Gd	PQ_RRRKKR/G	[39]
1998	Pakistan	H7N3	A/chicken/Pakistan/C‐1998/1998	—	PE_TPKRRKR/G	[40]
1999–00	Italy	H7N1	A/turkey/Italy/4580/1999	—	PE_IPKGSRVRR/G	[41]
2001–04	Pakistan	H7N3	A/chicken/Rawalpindi/Pakistan/NARC‐68/2002	—	PE_TPKRRKR/G	[40, 42]
2002	Chile	H7N3	A/chicken/Chile/184240–1/2002	—	PE_KPKTCSPLSRCRETR/G	[43]
2003	Netherlands, Belgium, Germany	H7N7	A/chicken/Netherlands/1/2003 A/chicken/Belgium/06775/2003 A/chicken/Germany/R28/2003	—	PE_IPKRRRR/G	[44] [45] [46]
2004	Canada	H7N3	A/chicken/Canada/AVFV2/2004	—	PE_NPKQAYRKRMTR/G	[47]
2004	USA	H5N2	A/chicken/Texas/298313/2004 (HPAIV molecularly pathotyped, isolate does not cause severe disease in chickens)	Am‐non‐gs/Gd	PQ_RKKR/G	[48]
2004	South Africa	H5N2	A/ostrich/South Africa/N227/2004	EA‐non‐gs/Gd	PQ_REKRRKKR/G	[49]
2005	North Korea	H7N7	A/chicken/North Korea/1/2005	—	^a^PE_IPKGRHRRPKR/G	[50]
2006	South Africa	H5N2	A/ostrich/South Africa/AI1091/2006	EA‐non‐gs/Gd	PQ_RRKKR/G	[51]
2007	Canada	H7N3	A/chicken/Saskatchewan/HR‐00011/2007	—	PE_NPKTTKPRPRR/G	[52]
2008	England	H7N7	A/chicken/England/1158–11406/2008	—	PE_IPKRKKR/G	[53]
2009	Spain	H7N7	A/chicken/Spain/6279–2/2009	—	^a^PE_LPKGTKPRPRR/G	[54]
2011	South Africa	H5N2	A/ostrich/South Africa/AI2114/2011	EA‐non‐gs/Gd	PQ_RRKKR/G	[55]
2012–13	Taiwan	H5N2	A/chicken/Taiwan/0101/2012	Am‐non‐gs/Gd	PQ_RKKR/G	[56, 57]
2012 ‐present	Mexico	H7N3	A/chicken/Jalisco/CPA1/2012	—	PE_NPKDRKSRHRRTR/G	[58]
2012	Australia	H7N7	A/chicken/New South Wales/3121–4/2012	—	^b^PE_IPRKRKR/G	[59, 60]
2013	Italy	H7N7	A/chicken/Italy/13VIR4527‐11/2013	—	PE_TPKRRERR/G	[61]
2013	Australia	H7N2	A/chicken/New South Wales/13–02811‐1/2013	—	^b^PE_IPRKRKR/G	[59, 62]
2015	England	H7N7	A/chicken/England/26352/2015	—	^a^PE_IPRHRKGR/G	[63]
2015	Germany	H7N7	A/chicken/Germany/AR1385/2015	—	^a^PE_IPKRKRR/G	[64]
2015–16	France	H5N1/x	A/chicken/France/150169a/2015	EA‐non‐gs/Gd	HQ_RRKR/G	[65]
2016	USA	H7N8	A/turkey/Indiana/16–001403‐1/2016	—	PE_NPKKRKTR/G	[66]
2016	Italy	H7N7	A/chicken/Italy/16vir1873/2016	—	^a^PE_LPKGRKRR/G	[67]
2016	Algeria	H7N1	Unknown	—	Unknown	[68]
2017–19	China	H7N9	A/chicken/Guangdong/SD008/2017	—	PE_VPKGKRTAR/G	[69]
2017	USA	H7N9	A/chicken/Tennessee/17–007147‐1/2017	—	PE_NPKTDRKSRHRRIR/G	[70]
2020	USA	H7N3	A/turkey/North Carolina/20–007949‐001/2020	—	^b^PE_NPKTDRKSRHRRIR/G	[71]
2020	Australia	H7N7	A/chicken/Lethbridge/20–02865‐0008/2020	—	^a^PE_IPGKREKR/G	[72, 73]
2023–24	South Africa	H7N6	A/chicken/South Africa/SA2310/2023	—	^a^PE_PPKGPRFRR/G	[74, 75]
2023	Mozambique	H7N6	A/chicken/Mozambique/857‐P3‐1_23VIR11699‐9/2023	—	^a^PE_PPKGPRFRR/G	[75, 76]
2024	Mexico	H5N2	Unknown	Unknown	Unknown	[77]
2024	Australia	H7N3	A/chicken/VIC/24–01759‐3/2024	—	^a^PE_IPGKRERR/G	[78]
2024	Australia	H7N9	A/chicken/VIC/10–17/2024	—	^a^PE_IPGKREKR/G	[79]
2024	Australia	H7N8	A/chicken/NSW/M24‐09739–19/2024	—	^a^Sequence pending	[80]
2024	Germany	H7N5	A/chicken/Germany‐NI/2024AI2733/2024	—	^b^PE_IPKKKKR/G	[81, 82]

*Note:* Table adapted from [59, 83, 84, 85].

^a^ HACS motif is not present in Table 4.

^b^ NA subtype associated with HACS motif is not present in Table 4.

The continued genetic evolution of gs/Gd‐lineage H5 HPAIVs lead to the formation of multiple clades and subclades [90]. Currently circulating clade 2.3.4.4 gs/Gd‐lineage H5 HPAIVs continue to diversify genetically, leading to the formation of provisional subclades, currently a‐h [91, 92]. Whilst gs/Gd‐lineage H5 HPAIVs were initially of the H5N1 subtype, currently circulating H5 HPAIVs are frequently detected with numerous other neuraminidase (NA) subtypes, such as N5, N6, and N8, and are referred to H5Nx viruses. It is currently unclear whether H5Nx HPAIVs more readily assort with diverse NA subtypes, than LPAIVs do, because there is less comprehensive data on LPAIVs. It has been hypothesised that H5Nx HPAIV emergence can be attributed to intense use of H5 vaccines driving expansion of antigenically distinct Nx subtypes in wild birds while supressing the H5N1 in vaccinated domestic birds [93].

The unprecedented prevalence, global spread, and evolution of H5N1 and H5Nx HPAIVs has heightened fears of their pandemic potential. In addition to the gs/Gd lineage, numerous lineages are extant in wild waterfowl and more rarely, poultry. These lineages also have the potential for zoonotic transmission. Although Eurasian non‐gs/Gd H5 and American non‐gs/Gd H5 lineages are well established, the subclades are less well defined than the gs/Gd‐lineage. Six North American H5 subclades, 1‐6, have been described [94]. North American H5's are more broadly categorised as ‘wild bird’, ‘live bird market’ or ‘Mexican’ lineage. Two major lineages of Eurasian H5 AIVs are described, early and contemporary, and the contemporary lineage is composed of three subgroups I‐III [95]. H7 AIVs viruses are classified into two major phylogenetic groups, the Eurasian and North American lineages [96]. Additional H7 clades include the Australian, equine, and ancestral H7 AIVs [96]. The North American H7 AIVs are classified into live bird market or wild bird subgroups [97].

A large body of evidence exists for the avian‐origin of human influenza viruses of numerous subtypes [98, 99], including the 1918 H1N1 [100], 1957 H2N2 [101, 102], 1968 H3N2 [103, 104], and 2009 H1N1 influenza virus pandemics [105]. AIVs continue to threaten the human population by providing an extensive gene pool for reassortment with circulating human strains, or by *in toto* infection from infected birds. Human infections with AIVs have been reviewed elsewhere [106], here we focus on some major H5 and H7 events. H5 and H7 HPAIV outbreaks have been devastating to the poultry population and industry, leading to the culling or death of millions of birds. Specifically, H5 HPAIV outbreaks between January 2005 and November 2022 resulted in the death and/or culling of 389 million birds. H7 HPAIV outbreaks for the same period resulted in the death and/or culling of 33 million birds (reviewed in [107]).

Because of numerous human cases since the early 2000s, H7 AIVs have become increasingly recognized for their pandemic potential. The first confirmed naturally‐acquired human infections with H7 HPAIV was reported in the Netherlands in 2003 [44]. Poultry workers in numerous outbreaks have been infected with H7 HPAIVs (Canada H7N3 2004; Mexico H7N3 2012; Italy H7N7 2013) [108, 109, 110]. The recent H7N9 epornitic that began in 2013 is of pandemic concern and continues to threaten the human population—between 2013 and 2019 there have been 1568 confirmed human cases with H7N9 and 616 deaths, equating to a 39.3% laboratory‐confirmed case fatality rate [111]. Whilst these infections were initially of the LPAIV pathotype, HPAIVs emerged in 2017 during the fifth wave of the epornitic [69] and have since infected humans. Since 2003 (up to March 2023), there have been over 120 documented cases of naturally acquired human H7 HPAIV infection (Table 2). Other notable H7 LPAIV infections in humans have occurred in the US and UK. In 1980, human infections with an avian‐origin seal H7N7 LPAIV in the US were reported [149]. In 2002, 2003, and 2016, H7N2 LPAIVs caused a handful of infections in the US in people that had high contact with infected animals [150, 151, 152, 153]. In 1996, a person in the UK was infected with H7N3 LPAIV following close contact with domestic ducks [154], and in 2006 (H7N3) and 2007 (H7N2) there were several cases of conjunctivitis and/or influenza‐like illness in poultry workers exposed to H7 LPAIV in the UK [155, 156]. More recently, the first detection of an H7N4 LPAIV human infection was reported [157].

**TABLE 2:** Highly pathogenic avian influenza virus human infections since 1996.^a^

Year	Country	Subtype	Representative isolate	# human infections^b^	Representative isolate HACS motif	References
1996–present	China—now global	H5N1	A/Hong Kong/156/1997 (Clade 0)	1031 total	PQ_RERRRKKR/G	[92, 112, 113, 114, 115, 116, 117, 118, 119, 120, 121, 122, 123, 124, 125, 126, 127, 128, 129, 130, 131, 132, 133, 134, 135, 136, 137, 138, 139, 140, 141, 142, 143, 144, 145]
		H5N1	A/Vietnam/1203/2004 (Clade 1)		PQ_RERRRKKR/G	
		H5N1	A/Cambodia/R0405050/2007 (Clade 1.1)	H5:	PQ_REGRRKKR/G	
		H5N1	A/Cambodia/V0606311/2011 (Clade 1.1.1)	8	PQ_REGRRKKR/G	
		H5N1	A/Cambodia/X0810301/2013 (Clade 1.1.2)		PQ_REERRKKR/G	
		H5N1	A/Indonesia/534H/2006 (Clade 2.1.2)	H5N1:	PQ_RERRRKKR/G	
		H5N1	A/Indonesia/6/2005 (Clade 2.1.3)	18 + 904	PQ_RERRRKKR/G	
		H5N1	A/Indonesia/5/2005 (Clade 2.1.3.2)		PQ_RESRRKKR/G	
		H5N1	A/Indonesia/NIHRD11771/2011 (Clade 2.1.3.2a)	H5N6:	Refer to GISAID	
		H5N1	A/Azerbaijan/001‐161/2006 (Clade 2.2)	94	PQ_GERRRKKR/G	
		H5N1	A/Egypt/N03072/2010 (Clade 2.2.1)		PQ_GERRRKKR/G	
		H5N1	A/Egypt/3300‐NAMRU3/2008 (Clade 2.2.1.1)	H5N8:	PQ_GERRRKKR/G	
		H5N1	A/Egypt/N04915/2014 (Clade 2.2.1.2)	7	Refer to GISAID	
		H5N1	A/Bangladesh/207095/2008 (Clade 2.2.2.1)		PQ_GERRRKKR/G	
		H5N1	A/Guangxi/1/2009 (Clade 2.3.2.1)		PQ_RERRRRKR/G	
		H5N1	A/Hubei/1/2010 (Clade 2.3.2.1a)		PQ_RERRRKR/G	
		H5N1	A/Hong Kong/5923/2012 (Clade 2.3.2.1b)		PQ_IERRRRKR/G	
		H5N1	A/Hong Kong/6841/2010 (Clade 2.3.2.1c)		PQ_RERRRKR/G	
		H5N1	A/Anhui/1/2005 (Clade 2.3.4)		PL_RERRRKR/G	
		H5N1	A/Hunan/1/2009 (Clade 2.3.4.1)		PL_RERRRKR/G	
		H5N1	A/Guizhou/1/2013 (Clade 2.3.4.2)		Refer to GISAID	
		H5N1	A/Viet Nam/HN31242/2007 (Clade 2.3.4.3)		PL_RERRRKR/G	
		H5N6	A/Sichuan/26221/2014 (Clade 2.3.4.4a)		Refer to GISAID	
		H5N8	A/Astrakhan/3212/2020 (Clade 2.3.4.4b)		PL_REKRRKR/G	
		H5N6	A/Sichuan/06681/2021 (Clade 2.3.4.4b)		PL_REKRRKR/G	
		H5N1	A/England/215201407/2021 (Clade 2.3.4.4b)		Refer to GISAID	
		H5N1	A/Colorado/18/2022 (Clade 2.3.4.4b)		Refer to GISAID	
		H5N6	A/Hubei/29578/2016 (Clade 2.3.4.4d)		Refer to GISAID	
		H5N6	A/Hunan/55555/2016 (Clade 2.3.4.4g)		Refer to GISAID	
		H5N6	A/Guangdong/18SF020/2018 (Clade 2.3.4.4h)		Refer to GISAID	
		H5N1	A/Hong Kong/378.1/2001 (Clade 3)		PQ_RERRRKKR/G	
		H5N1	A/Beijing/01/2003 (Clade 7)		PQ_REGRRKKR/G	
2003	Netherlands	H7N7	A/Netherlands/219/2003	89	PE_IPKRRRR/G	[146]
2004	Canada	H7N3	A/Canada/444/2004	2	PE_NPKQAYQKQMTR/G	[108]
2012	Mexico	H7N3	A/Mexico/InDRE7218/2012	2	PE_NPKDRKSRHRRTR/G	[109]
2013	Italy	H7N7	A/Italy/3/2013	3	PE_TPKRRERR/G	[110]
2017‐19	China	H7N9	A/Guangdong/Th008/2017	32	PE_VPKRKRTAR/G	[111, 147, 148]

^a^ Laboratory‐acquired infections not included.

^b^ As of September 2024.

In 1997, H5N1 HPAIV human infections were first detected. Of the confirmed 18 H5N1 human infections, 6 were fatal [112, 158, 159]. Between 2003 and September 2024, there have been 904 laboratory confirmed human H5N1 infections and 464 deaths, equating to a 51.3% case fatality rate [113] (Table 2). All laboratory confirmed H5 HPAIV human infections to date are associated with the gs/Gd‐lineage. Whilst gs/Gd‐lineage human infections were initially caused by H5N1 HPAIVs, recent human gs/Gd‐lineage H5 infections have been predominately caused by H5N6 HPAIVs, although human infections with H5N1 and H5N8 HPAIVs have been recently reported [113, 114, 115, 116, 117, 118, 119, 120, 121, 122, 123, 124, 125, 126, 127, 128, 129, 130, 131, 132, 133, 134, 135, 160, 161, 162, 163]. Recently, the first laboratory‐confirmed human fatality with Eurasian non‐gs/Gd lineage H5N2 AIV has been reported [164]. To predict the emergence of HPAIVs and prevent spill‐over events that devastate poultry and pose a pandemic threat to human populations, it is crucial to understand the molecular attributes that drive the evolution of HPAIVs.

## 2 Biology of Influenza a Virus

Influenza viruses belong to the *Orthomyxoviridae* family, and are characterised by their single‐stranded, negative sense, segmented RNA genome. The *Orthomyxoviridae* family contains nine genera, of which influenza viruses are present in four of these genera, (1) *Alphainfluenzavirus*, (2) *Betainfluenzavirus*, (3) *Deltainfluenzavirus*, and (4) *Gammainfluenzavirus* [165]. *Alphainfluenzaviruses* (Influenza A) can infect a wide variety of hosts, including most mammals and avian species, whereas influenza viruses in *Betainfluenzavirus*, *Deltainfluenzavirus*, and *Gammainfluenzavirus* genera have limited host specificity.

Influenza A viruses contain eight gene segments that encode their cognate proteins: polymerase basic 2 (PB2), polymerase basic 1 (PB1), polymerase acidic (PA), HA, nucleoprotein (NP), NA, matrix (M, protein: M1), and non‐structural (NS, protein: NS1). Alternative splicing of the M and NS genes encodes matrix 2 and the more recently identified M42 [166, 167], and non‐structural 2 (also known as nuclear export protein) proteins, respectively. An additional M transcript has been reported, mRNA_3,_ [168] although to date a translated product has not been detected. Numerous strain‐specific accessory proteins may also be expressed, including PB1‐F2 [169], PB1‐N40 [170], PA‐X [171], PA‐N182, PA‐N155 [172], NS3 [173], and NSP [174, 175].

Influenza A viruses are divided into numerous subtypes based on genetic and antigenic characteristics of the surface glycoproteins, HA and NA [8]. Currently, there are 19 HA (H1‐19) and 11 NA (N1‐11) subtypes. AIVs can be any of the HA1‐16/H19 and N1‐9 subtypes, and most combinations of these subtypes have been isolated from wild aquatic birds [3, 176]. Whilst H17 and H18 influenza‐like viruses have been described, these subtypes are quite divergent from the H1‐16 subtypes and have only been detected in bats [177, 178].

**FIGURE 1:**
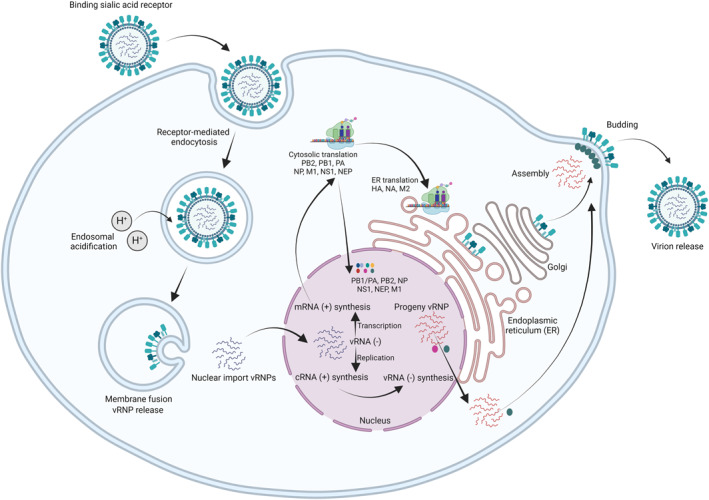
Schematic of Influenza A virus replication cycle. Upon HA binding to sialic acid receptors, influenza virions are internalised by receptor‐mediated endocytosis [179]. Endosomal acidification triggers HA to undergo an irreversible conformational rearrangement and insert the fusion peptide into the host cell and initiate membrane fusion [180, 181, 182, 183]. Endosomal acidification also leads to an influx of protons via destabilised M2 tetramers which allows the vRNPs to disassociate from the matrix protein [184]. The eight vRNPs are then released and trafficked to the nucleus via importin‐α‐importin‐β nuclear import pathway [185]. Transcription and replication of the viral genome by the RNA‐dependent RNA polymerase generates mRNA transcripts (+) and complementary RNA (+) followed by progeny viral genome (−), respectively. mRNA is exported out of the nucleus where cytosolic translation of PB2, PB1, PA, NP, M1, NS1 and NEP occurs. Newly synthesised PB1/PA heterodimers, PB2, and NP are imported into the nucleus via importin‐α‐importin‐β nuclear import pathway for secondary transcription and replication (reviewed in [186]). NS1 is also imported into the nucleus to suppress the host antiviral response [187] and newly synthesised NEP and M1 are imported into the nucleus where they are involved in the export of progeny vRNPs. Progeny vRNPs and M1 protein are then trafficked to the plasma membrane via RAB11A [188] for viral assembly. Whilst initial translation of HA, NA, and M2 occurs cytosolically, the signal peptide directs the ribosome with nascent HA, NA, or M2 to the endoplasmic reticulum for translation. HA, NA, and M2 are then trafficked through the Golgi before assembly at the plasma membrane followed by virion budding (reviewed in [186]). Figure created with BioRender.com.

The HA and NA surface glycoproteins play crucial roles in the IAV life cycle (Figure 1). HA recognises sialic acid receptors on the surface of host cells and the linkage of sialic acid to penultimate galactose on carbohydrate side chains contributes to IAV host range. AIVs preferentially bind α‐2,3 linked sialic acids whereas human influenza viruses preferentially bind α‐2,6 linked sialic acids [189]. However, viruses that exhibit dual sialic acid receptor specificity have been identified [189, 190, 191, 192, 193]. There may be some variation among avian species for moieties of each sialic acid structure and expression patterns as well [194, 195, 196, 197, 198, 199]. The entry receptor for H17 and H18 bat influenza viruses is major histocompatibility complex class II, human leucocyte antigen DR α chain [200] which is atypical for influenza viruses. However, major histocompatibility complex class II receptor usage has been recently described for human and avian H2 [201] and H19 avian influenza viruses [202]. The receptor preference for the H9N2 influenza virus isolated from an Egyptian bat remains the α‐2,3 linked conformation of the canonical sialic acid receptor [203]. Upon receptor binding, influenza virions are internalized via receptor‐mediated endocytosis [179] or micropinocytosis [204]. Whilst HA binds to sialic acid receptors, evidence suggests that endocytosis signal transduction is mediated by a functional receptor including N‐linked glycoprotein [205], the receptor tyrosine kinases epidermal growth factor receptor and c‐Met [206], DC‐SIGN [207, 208] and L‐SIGN [208], nucleolin [209], Ca_V_1.2 [210], free fatty acid receptor 2 [211], transferrin receptor 1 [212], and metabotropic glutamate receptor subtype 2 [213]. Endosomal acidification triggers HA to undergo a conformational rearrangement that causes the HA fusion peptide to be inserted into the host membrane and initiate membrane fusion [180, 181, 182, 183]. Endosomal acidification also protonates histidine‐37 of the M2 ion channel, destabilising the M2 tetramer and opening the tyrosine‐41 gate. The proton influx allows the ribonucleoproteins to dissociate from the matrix protein [184]. The eight viral ribonucleoproteins (vRNPs) are subsequently trafficked to the nucleus, where transcription (vRNA (−) → mRNA (+) and replication (vRNA (−) → cRNA (+) to vRNA (−)) of the influenza virus genome occurs (reviewed in [186]). Newly synthesised vRNPs and proteins are transported to the surface of the host cell membrane, where viral budding is initiated [214]. Following bud formation and closure, the glycolytic activity of NA enables virus release from sialic acid receptors present at budding sites of infected cells. Inhibition of NA enzymatic activity leads to defective viral release, resulting in aggregation of progeny virions at the site of budding [215, 216]. Inhibition of influenza virus NA activity is the mechanism‐of‐action of the US Food and Drug Administration currently approved anti‐influenza antivirals, Tamiflu (oseltamivir phosphate), Relenza (zanamivir) and Rapivab (peramivir) [217, 218, 219].

Characterisation of HA and NA density on the surface of influenza virions have revealed some diversity in the expression ratio, ranging from of 3:1 up to 18:1 [220]. As HA and NA activity is critical for host cell binding and release, variation in the expression ratio may modulate the functional balance, leading to altered virion entry or egress dynamics. A second NA sialic binding site present in most AIVs contributes to HA and NA functional balance by modulating NA receptor catalysis, particularly for multivalent substrates [221, 222] in addition to influencing receptor binding dynamics [222]. A follow up study demonstrated that substitutions in the second NA sialic acid binding site of H5 AIVs are associated with compensatory HA substitutions that modulate receptor binding properties [223]. Interestingly, HA also has a second (vestigial) HA sialic acid binding site [224], although the functional role of this additional site is unclear.

## 3 Proteolytic Cleavage of Influenza Haemagglutinin

Infectivity of influenza viruses is dependent on cleavage of the HA surface glycoprotein [225, 226]. The HACS motif is located on a solvent exposed loop [227] and cleavage of mature HA_0_ into two disulphide linked subunits, HA_1_ and HA_2_, enables the low pH induced conformational rearrangement required for fusion with the host cell membrane to occur. The number of basic amino acids in the HACS motif modulates the family of activating proteases that cleaves it, and it is also a prime determinant of AIV pathogenicity in gallinaceous species [8, 83].

Nomenclature of substrate subsites are based on that described by Schechter and Berger [228], where residues N‐terminal of the scissile bond are defined as P1 (and S1 for the corresponding enzyme subsite) and those C‐terminal as P1’ (and S1′ for the corresponding enzyme subsite). The subsite numbering increases with distance from the scissile bond.

### 3.1 Cleavage of LPAIV Haemagglutinin by Proteases With Monobasic Specificity

Low pathogenicity avian influenza viruses typically have either one or two non‐consecutive basic amino acids in the HACS motif (Table 3) and are cleaved by trypsin and trypsin‐like proteases (peptidase family S1, subfamily S1A) [225, 229, 230, 231, 232, 233]. Trypsin cleaves C‐terminal to arginine (Arg) or lysine (Lys), with cleavage of Arg 2‐10 fold more efficient then cleavage of Lys [234]. The monobasic substrate specificity of the major LPAIV activating enzyme, trypsin, is determined by an acidic residue, aspartic acid‐189, that is present at the base of trypsin's S1 binding pocket [234]. Trypsin is synthesised as a zymogen (trypsinogen) in pancreatic acinar cells that is then secreted into the small intestine where it is processed into the active form by enteropeptidase or by trypsin itself [234]. The presence of activated trypsin in the intestinal tract supports intestinal replication of LPAIV, which is a hallmark of LPAIV infection in wild waterfowl [235]. Trypsin has also been detected in numerous other tissues including the respiratory tract, gastrointestinal tract, and kidneys [236]. IAVs harbouring monobasic HACSs motif can also be activated by numerous trypsin‐like proteases including plasmin [230] and its derivative mini‐plasmin [237], factor Xa [231, 238], tryptase Clara [232], mast cell tryptase [233], tryptase TC30 [239], human airway trypsin‐like protease [229], transmembrane serine protease 2 [229], transmembrane serine protease 4 [240], transmembrane serine protease 11A [241], transmembrane serine protease 11E (also known as DESC1) [242], matriptase [243], kallikrein 5 and 12 [244], mosaic serine protease large‐form (MSPL) [242], and N6‐dependent cleavage by thrombin [245]. The expression of multiple trypsin and trypsin‐like proteases with the ability to cleave HA at mucosal surfaces of the respiratory and gastrointestinal tracts contributes to tissue tropism of LPAIVs, restricting replication of influenza viruses with monobasic HACS motifs to these locales.

**TABLE 3:** Consensus haemagglutinin cleavage site motif of avian H1‐H16 LPAIVs and bat‐origin H17‐18 IAVs.

	Subtype	Consensus HACS motif	Number of basic amino acids in HACS motif
Avian LPAIV	H1	PS_IQSR/G	1
	H2	PQ_IESR/G	1
	H3	PE_KQTR/G	2
	H4	PE_KATR/G	2
	H5	PQ_RETR/G	2
	H6	PQ_IETR/G	1
	H7	PE_IPKGR/G	2
	H8	PS_IEPK/G	1
	H9	PS_RSSR/G	2
	H10	PE_VVQGR/G	1
	H11	PA_IATR/G	1
	H12	PQ_AQNR/G	1
	H13	PA_ISNR/G	1
	H14	PD_KQTK/G	2
	H15	PE_KIHTR/G	3
	H16	PS_SINER/G	1
Bat	H17	PQ_MEGR/G	1
	H18	PI_KETR/G	2

### 3.2 Cleavage of HPAIV Haemagglutinin by Proteases With Polybasic Specificity

**FIGURE 2:**
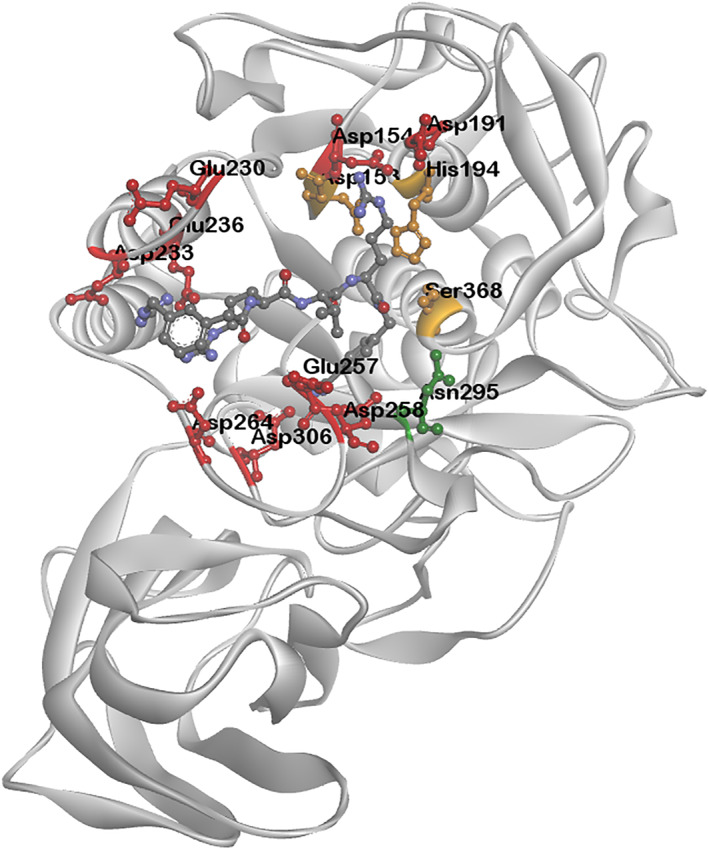
Protein homology model of chicken furin complexed with inhibitor. Protein homology model of chicken furin (NP_990046.1) based on the crystal structure of 4OMC [248] was generated using SWISS‐MODEL [249] and modified using BIOVIA Discovery Studio (Dassault Systèmes). Acidic, negatively charges amino acids lining the active site cleft (substrate binding domain) are shown in red: S1, Asp258 and Asp306; S2, Asp154 and Asp191; S3, Glu257; S4, Glu236 and Asp264; S5, Asp233; S6, Glu230 and Asp233. Catalytic triad (Ser368, His194, Asp153), orange. Oxyanion hole (Asn295), green [247, 248]. Inhibitor is shown within the active site cleft.

In contrast to LPAIVs, HPAIVs contain multiple basic amino acids in the polybasic HACS (pHACS) motif, facilitating cleavage by the ubiquitously expressed proprotein convertases, most notably, furin (peptidase family S8, subfamily S8B). Understanding the biochemical specificity of furin provides a mechanistic basis as to why HPAIVs that harbour multiple basic amino acids in the pHACS motif seem to occupy a fitness peak and therefore are selected for in naturally occurring populations. Furin cleaves C‐terminal to the consensus motif Arg‐Xaa‐Arg/Lys‐Arg [246]. Crystallography studies identified a patch of acidic amino acids lining furin's active site cleft (substrate binding domain) that imparts a negative electrostatic potential and preferentially interacts with substrates that harbour multiple basic (positively charged) residues [247, 248] (Figure 2). Subsites S1, S2, and S4 in the active site cleft favour Arg and Lys residues. Specifically, furin's strict preference for Arg at P1 is due the ability of the side chain of Arg to optimally occupy the S1 pocket. Side chains from other amino acids, including Lys, can occupy the S1 pocket, however the alignment is suboptimal. The charge and geometry of S2 are optimal for Lys at P2, although Arg can be accommodated. The sidechain of Arg is optimally accommodated within the S4 cleft, although side chains from other amino acids can be accommodated [247]. Similar to P1, furin prefers Arg over Lys at P4, although the requirement at this subsite is not as stringent. Subsite requirements at P3, P5, and P6 are not as stringent as P1, P2, or P4. However, due to the negative electrostatic potential of the active site cleft, basic residues are favoured. Biochemical studies with a tetra‐L‐arginine furin peptide inhibitor revealed that increasing the number of basic Arg residues in the inhibitor (penta‐L‐arginine and hexa‐L‐arginine) increased the K_*i*_ by 6.4x and 56.2x, respectively, suggesting that P5 and P6 contribute to optimal substrate interactions with furin [250], which was confirmed by recent structural studies [251]. This preference for basic residues may extend to subsites P7 and P8 [247]. A reduction in the number of positively charged, basic residues in furin substrates may lead to suboptimal presentation of the scissile bond to the active site [247], driving the preference for substates that contain multiple basic amino acids. Finally, the amino acid occupying P1’ (which is usually glycine (Gly)) has been shown to affect cleavage efficiency of HPAIVs [252], which may also be attributed to suboptimal scissile bond presentation or steric hindrance preventing optimal engagement with activating protease.

Furin is a membrane‐bound, ubiquitously expressed enzyme within the constitutive secretory pathway that is synthesised as a zymogen (profurin). A series of autocatalytic events *en route* to the *trans*‐Golgi network activates furin [253], where it predominantly resides, although furin also cycles between the *trans*‐Golgi network, endosomes and the cell surface [254, 255]. Additionally, a secreted form of furin has also been described [256]. Newly synthesised HA trimers are translocated through the constitutive pathway where they are cleaved by furin in the *trans*‐Golgi network and stabilised in the native conformation by M2‐mediated regulation of the intra‐compartmental pH [257]. The ability of HPAIV to be cleaved by ubiquitously expressed proteases facilitates systemic replication of HPAIV in birds. Interestingly, chicken and duck furin are conserved functionally and have comparable tissue expression (at the mRNA level), suggesting that differences in HPAIV pathogenesis in these species is unlikely to be mediated by furin [258].

Highly pathogenic avian influenza virus HA can also be activated by proprotein convertase (PC) 5/6 (PC5/6), although PC5/6 seems to be more selective than furin [259]. Alternate splicing of PC5/6 yields two isoforms; the ubiquitously expressed soluble form PC5/6A is transported to the cell surface via the regulated secretory pathway [260] where it is anchored to heparin sulphate proteoglycans [261], and the tissue restricted membrane‐bound PC5/6B form that is localised within the constitutive secretory pathway [260]. Like all proprotein convertases, PC5/6 is synthesised as a zymogen, and in a manner similar to furin, PC5/6B undergoes a series of autocatalytic events *en route* to the *trans*‐Golgi network where it predominantly resides [260], although PC5/6B also cycles between the *trans*‐Golgi network, endosomes, and the cell surface [262]. Unique among the proprotein convertases, the final PC5/6A autocatalytic event occurs at the cell surface [263]. Initial studies suggested that PC5/6A cleaves monobasic and dibasic substrates and the presence of Arg at P4 is not a requirement [264]; however, subsequent substrate cleavage analysis suggests that the absence of Arg at P4 abrogates cleavage by PC5/6A and PC5/6B [265]. Although currently lacking, crystal structures of PC5/6A and PC5/6B bound to a substrate or inhibitor would provide further insights about the specificity of these enzymes.

The type II transmembrane serine protease, MSPL, and its splice variant, transmembrane serine protease 13, can also activate HPAIV HA [266, 267]. MSPL belongs to the type II transmembrane serine protease family and MSPL mRNA transcripts are most abundantly expressed in the lung, spleen, pancreas, placenta, and prostate in humans [267, 268]. The expression pattern of TEMPSS13/MSPL in birds is currently unknown. Biochemical studies suggest that the optimal recognition motif of MSPL is Lys‐Lys‐Lys‐Arg, although variations meeting the consensus motif Arg/Lys‐Xaa‐Arg/Lys‐Arg are efficiently cleaved [266]. Recently, the crystal structure of MSPL bound to a furin inhibitor was solved [269] providing insights about the substrate specificity of MSPL. The P1 Arg side optimally occupies the deep S1 pocket in a manner typical for trypsin and trypsin‐like proteases. However, the presence of a unique acidic‐residue rich 99‐loop in MSPL favours interactions with P2 Lys and P4 Arg sidechains [269]. Although structurally MSPL resembles members of trypsin family of serine proteases (S1 family), this 99‐loop which is unique to MSPL favours interactions with polybasic substrates [269].

Lastly, cleavage analysis of peptides mimicking HPAIVs pHACS motif suggests that some proteases with monobasic specificity, trypsin, matriptase and plasmin, can also cleave the HPAIV HACS motif [270].

## 4 Mechanisms Driving Expansion of the Haemagglutinin Cleavage Site Motif

Multiple epidemiologic, sequencing, and phylogenetic studies have provided robust evidence for the emergence of HPAIVs from LPAIV precursors [64, 271, 272, 273, 274, 275, 276, 277]. The emergence of HPAIVs from LPAIV precursors has been proposed to occur by three mechanisms: (1) substitutions due to low polymerase fidelity, (2) insertions/duplications from RNA secondary structure‐mediated RNA dependent RNA polymerase (RdRp) slippage [276, 278, 279], and (3) non‐homologous recombination with viral or host RNA [275, 280, 281].

The low replication fidelity of IAV RdRp resulting in substitutions in the HACS motif is one hypothesis for the mechanism driving the molecular evolution of the HACS motif, although direct evidence remains elusive, and it is likely that other mechanisms are driving the molecular evolution of the HACS motif. Of the eight influenza gene segments, the overall substitution (mutation) rate of HA is the highest at 3.92 × 10^−3^ substitutions/site/year. Subtype specific analysis revealed increased HA substitution rates for H7N2, H7N7, H5N1, and H5N2 subtypes. H5N2 viruses exhibited the highest substitution rate of 5.33 × 10^−3^ substitutions/site/year, which increased to 8.39 × 10^−3^ substitutions/site/year for H5N2 chicken isolates [282].

Stem‐loop mediated polymerase slippage is another proposed mechanism describing insertions that encode additional basic amino acids in the HACS motif and HPAIV genesis. This mechanism describes how the RdRp encounters RNA stem‐loop secondary structure within HACS motif region that causes the RdRp to pause, slip, and then continue reading the template strand [278, 279]. RdRp slippage inserts additional purines into the already purine rich H5/H7 HACS motif [283] (duplication or stepwise accumulation), ultimately expanding the HACS motif to include additional basic amino acids. Specifically, it has been hypothesised that the stem reforms around the RdRp, impeding processivity and promoting insertions [284]. Numerous studies describe insertions or duplication events in the H5 and H7 HACS motif [278, 279, 285, 286, 287, 288], which positions basic amino acids at crucial subsites. Evidence for RdRp mediated insertions in the HACS motif in naturally occurring HPAIV infection has been recently described. Sequence analysis of a viral population from H5 HPAIV infected Ostrich revealed RdRp slippage incorporated up to 11 adenines (uracil in vRNA) (encoding multiple Lys) into the region encoding the HACS motif [276]. HPAIV quasispecies also contained substitutions and/or insertions in the HACS motif, providing a diverse mutational space to sample [276]. Similarly, multiple HACS motifs containing various number of Lys insertions were detected during the 2008 H5 UK epizootic [59]. The HPAIV pHACS motif encoding multiple basic amino acids are predicted to have a bulged looped structure, which is absent from LPAIVs [276]. Interestingly, the mid‐length HACS motif PQ_REKR/G is also predicted to have a bulged RNA secondary structure reminiscent of HPAIV [276], which likely promotes RdRp slippage events. This hypothesis is supported by an additional study which revealed RdRp mediated incorporation of additional purines into the HACS motif enlarges the stem‐loop structure which promotes incorporation of additional purines [289]. A cRNA enlarged loop size [290] and consecutive adenines are crucial for promoting insertions [290, 291]. This described mechanism for HACS expansion resembles that of IAV mRNA polyadenylation, a similar process by which IAV RdRp slippage occurs upon encountering 5′ RNA stem‐loop secondary structure which causes it to insert a string of adenines [292, 293]. In both instances, interruption of transcription by RNA secondary structure leads to incorporation of additional nucleotides.

Recombination events with viral or host RNA seem to be a hallmark of H7 HACS motif expansion, although substitutions and insertions do occur. Interestingly, H7 HPAIV genesis through non‐homologous recombination is predominately associated with the N3 subtype (Table 4). The 2004 HPAIV epizootic in Canada occurred following non‐homologous recombination between the HA and M gene segments of low pathogenic precursors. This intersegmental recombination lead to a 21 nucleotide insertion, altering the HACS motif from PE_NPKTR/G to PE_NPKQAYRKRMTR/G (insertion underlined) [47, 275] that ultimately positioned basic amino acids at subsites P4, P5 and P6 which would favour interactions with enzymes with polybasic specificity. Similarly, a recombination event between HA and NP genes contributed to the emergence of the 2002 HPAIV epizootic in Chile [280]. A 30‐nucleotide insertion modified the LPAIV HACS from PE_KPKTR/G to PE_KPKTCSPLSRCRKTR/G or PE_KPKTCSPLSRCRETR/G, again positioning basic amino acids at crucial subsites. Recombination events with host RNA [52], and most frequently, host 28S ribosomal RNA [58, 70, 71, 281], have also been detected in H7 HPAIVs. Microhomology between foreign and HA RNA has been identified for some H7 HPAIVs, and recognition of micro‐homologous (palindromic) sequences may drive recombination events, although a mechanism by which this occurs remains elusive [281]. It has been hypothesised that recombination events may involve small nucleolar RNA binding sites [294]. Recombination events with 28S ribosomal RNA situated basic amino acids at crucial subsites (Table 4), suggesting that recombination events with viral and host RNA that lead to expansion of the HACS motif serves to optimise interactions with available proteases that exhibit polybasic specificity. Intersegmental recombination between H7 genes [295] or host 28S ribosomal RNA [296], and substitutions and insertions in the nucleotide sequence that encodes the HACS motif [297] have occurred experimentally following virus passage under conditions that contain alternate activating proteases, or when trypsin is absent completely. Furthermore, HPAIVs have been preferentially selected in older embryonated chicken eggs (E12 and E14) [298], which was later suggested to occur because of differential protease and/or because of interferon activity in older embryonated chicken eggs [299]. Recently, the first evidence for intersegmental non‐homologous recombination event between H5 HA and NP genes has been demonstrated experimentally [300].

**TABLE 4:** Frequency of H7 haemagglutinin cleavage site motifs and associated neuraminidase subtypes.

	H7 HACS motif^a^	Neuraminidase subtype											HACS motif length	Number of basic amino acids in HACS motif	Frequency	HPAIV^b^
		N1	N2	N3	N4	N5	N6	N7	N8	N9	Mixed	H7				
Extended	PE_NSTHKQLTHHMRKKR/G							N7					15	8	14	—
	PE_APAHKQLTHHMRKKR/G							N7					15	8	3	—
	PE_NSIHKQLTHHMRKKR/G							N7					15	8	2	—
	PE_KPKTCSPLSRCRKTR/G			N3									15	6	3	Y
	PE_KPKTRAPFGTKSQRR/G			N3									15	6	1	U
	PE_KPKTCSPLSRCRETR/G			N3									15	5	5	Y
	PE_NPKPEAPFQREAQRR/G			N3									15	4	1	U
	PE_NPKPEAPFQPEAQRR/G			N3									15	3	1	U
	PE_NPKPDAPLYGNPKTR/G			N3									15	3	1	U
	PE_NPKPDGPVPGEPQTR/G			N3									15	2	1	U
	^f^PE_NPKTDRKSRHRRIR/G									N9			14	8	12	Y
	PE_NPKDRKRRHRRTR/G			N3									13	9	2	Y
	PE_NPKDRKSRHRRTR/G			N3									13	8	28	Y
	PE_NPKDRKNRHRRTR/G			N3									13	8	7	Y
	PE_NPKGKKSRHRKTR/G			N3									13	8	7	Y
	PE_NPKGRKSRHRKTR/G			N3									13	8	2	Y
	PE_NPKDRKGRHRRTR/G			N3									13	8	1	Y
	PE_NPKDKKSRHRRTR/G			N3									13	8	1	U
	PE_NSKDMKSRHRKTR/G			N3									13	7	2	Y
	PE_NPKDWKSRHRRTR/G			N3									13	7	1	Y
	PE_NPKQAYRKRMTR/G			N3									12	5	3	Y
	PE_NPKQAYQKRMTR/G			N3									12	4	1	Y
	PE_NPKQAYQKQMTR/G			N3									12	3	1	Y
	PE_NPKTTKPRPRR/G			N3									11	5	1	Y
	PE_IPKKKKKKR/G							N7					9	7	1	Y
	PE_TPKKKKKKR/G							N7					9	7	1	Y
	PE_VPKRKRTAR/G		N2							N9	Mixed		9	5	57	Y
	PE_VPKRRRTAR/G			N3						N9			9	5	2	Y
	PE_IPKGSRVRR/G	N1											9	4	34	Y
	PE_VPKGKRTAR/G									N9			9	4	19	Y
Extended (continued)	PE_VPKGKRIAR/G									N9			9	4	1	Y
	PE_IPKGKRTAR/G									N9			9	4	1	U
	PE_LPKKRRKR/G							N7					8	6	3	Y
	PE_PSKKRKKR/G	N1											8	6	2	Y
	PE_TPKRKRKR/G			N3									8	6	2	Y
	PE_IPKKRKKR/G							N7					8	6	1	Y
	PE_IPKKREKR/G							N7					8	5	5	Y
	PE_NPKKRKTR/G								N8				8	5	5	Y
	PE_TPKRRERR/G							N7					8	5	1	Y
	PE_TPKRRKR/G			N3									7	5	18	Y
	PE_IPKRRRR/G							N7					7	5	6	Y
	^d^PE_IPRKRKR/G			N3	N4								7	5	4	Y
	^e^PE_IPKKKKR/G			N3				N7					7	5	3	Y
	PE_IPRRRKR/G				N4								7	5	2	Y
	PE_IPKRKKR/G							N7					7	5	1	Y
	PE_TPKRRRR/G			N3									7	5	1	Y
Mid‐length	PE_IPKRRR/G	N1											6	4	1	Y
	PE_KPKKR/G		N2										5	4	47	N
	PE_KPKPR/G		N2										5	3	188	N
	PE_KPKTR/G		N2	N3			N6	N7		N9	Mixed		5	3	36	N
	PE_KPRTR/G						N6						5	3	15	N
	PE_IPKRR/G			N3			N6	N7					5	3	5	N
	PE_QPKRR/G						N6						5	3	3	N
	PG_VPRKR/G		N2										5	3	1	N
	PE_IPKKR/G							N7					5	3	1	N
	PE_IPRKR/G						N6						5	3	1	N
Short	PE_IPKGR/G	N1	N2	N3	N4	N5	N6	N7	N8	N9	Mixed	H7	5	2	1224	N
	**R**E_IPKGR/G									N9			5	2	1	N
	PE_NPKTR/G	N1	N2	N3	N4	N5	N6	N7	N8	N9	Mixed	H7	5	2	756	N
Short (continued)	PE_LPKGR/G	N1	N2	N3	N4	N5	N6	N7	N8	N9			5	2	104	N
	PE_TPKGR/G	N1		N3			N6	N7		N9	Mixed		5	2	85	N
	PE_VPKGR/G	N1						N7	N8	N9	Mixed		5	2	22	N
	PE_PPKGR/G			N3			N6	N7		N9			5	2	12	N
	PE_NPKPR/G		N2										5	2	12	N
	PE_IPKGK/G							N7					5	2	6	N^c^
	PE_NPKAR/G			N3				N7					5	2	6	N^c^
	PE_LPKGK/G	N1											5	2	5	N
	PE_IPKER/G	N1						N7	N8				5	2	4	N
	PE_IPRGR/G						N6	N7					5	2	3	N
	PE_GPKER/G							N7					5	2	3	N
	PE_NSKTR/G	N1		N3									5	2	2	N^c^
	PE_SPKGR/G							N7					5	2	1	N
	PE_SPKTR/G								N8				5	2	1	N
	PE_SPKAR/G							N7					5	2	1	N^c^
	PE_DPKTR/G						N6						5	2	1	N^c^
	PE_NPKTK/G										Mixed		5	2	1	N^c^
														**TOTAL**	**2819**	

^a^ Basic amino acids flanking the defined HACS motif in boldface.

^b^ Y = yes. N = no. U = Unknown. Pathotype information not publicly available. − = not applicable (equine).

^c^ Conforms to molecular definition of LPAIV.

^d^ Present in 2012 H7N7 and 2013 H7N2 HPAIVs.

^e^ Present in 2024 H7N5 HPAIV.

^f^ Present in 2020 H7N3 HPAIV—see Table 1.

Whilst numerous studies describe insertions in the HACS motif of naturally occurring and experimentally derived viruses, unequivocal evidence for what drives expansion of the HACS motif remains elusive. Specifically, why is expansion of HACS motif leading to (naturally occurring) pathotype conversion seemingly limited to H5 and H7 subtypes only despite experimental evidence that other subtypes can support a HPAIV phenotype? [301, 302, 303] What conditions promote expansion of the HACS motif? Why does HACS motif expansion occur in gallinaceous poultry and seemingly not favoured in wild aquatic birds? Interestingly, HPAIV has a lower infectious dose in ducks (*Anas platyrhynchos*), chickens (*Gallus gallus domesticus*) and turkeys (*Meleagris gallopavo domesticus*) [304, 305], which suggests that infectivity is a selection factor. These aspects of influenza evolution remain of great interest and despite decades of research, gaps in our knowledge remain. Comparative and interdisciplinary studies combining molecular biology, biochemical, virological, and bioinformatics approaches may shed light on mechanisms driving expansion of the HACS motif and pathotype conversion.

## 5 Sequence Diversity of H5 and H7 Avian Influenza Virus HA Cleavage Site Motif: A Prime Determinant of Pathogenicity

Low pathogenicity avian influenza viruses have been isolated from more than 100 species of wild birds [89], but dabbling ducks (*Anatidae* family) and some shorebirds (*Scolopacidae* and *Laridae* families) are considered the natural hosts and serve as reservoirs that harbour a diverse influenza gene pool for other avian and mammalian species. The widespread prevalence of LPAIVs (that generally have one or two non‐consecutive basic amino acids in the HACS motif, Table 3) in wild aquatic birds suggests that LPAIVs seem to occupy a fitness peak in their ecological niche. The tissue tropism of LPAIV is also predominantly in the alimentary tract, in contrast to other species where replication is mainly in the upper respiratory tract.

Conversion events from low to high pathogenicity are associated with expansion of the HACS motif to include multiple basic amino acids and occur during circulation in non‐natural host species (*Pangalliformes* clade) suggesting some selective pressure is present. Emergence of HPAIVs have occurred in all major lineages of H5 and H7 AIVs. Although the highly pathogenic phenotype has evolved naturally in the H5 and H7 subtypes only, isolates of the H4 [306], H7 [307] and H9 [308] subtypes with > 2 basic amino acids in the HACS motif have been described. Also an H10 AIV with an IVPI > 1.2 has been reported despite not having a pHACS [309]. Experimental studies have identified that other subtypes including H2, H4, H6, H8, H9 and H14 can support a highly pathogenic phenotype [301, 302, 303, 310] whereas H1, H3, H10, H11 and H15 has not been shown to support high pathogenicity [301, 311, 312]. Finally, steric hindrance resulting from a nearby carbohydrate can modulate pathogenic phenotype outcomes [27, 48]. This suggests that other genetic features not in the HACS can influence the highly pathogenic phenotype of AIVs.

Building upon previous analysis of naturally occurring H5 and H7 HACS motifs [84, 313], the molecular composition and frequency of H5 and H7 HACS motifs were analysed to provide trends and insights about the relative functional fitness of particular HACS motif. Here, full length H5 and H7 HA protein sequences were obtained from the Influenza Research Database [314] and sequences with ambiguous base calls, laboratory‐generated, and duplicate sequences were omitted. The exception to this was gs/Gd‐lineage clade 8 sequence analysis, as full‐length sequences were unavailable, so analysis was performed on partial sequences that contained full‐length HACS motifs. We note that there may be sample bias to some lineages due to differences in surveillance programs and focus on lineages from domestic species. HACS motifs present in H5 Eurasian (850 sequences), H5 North American (319 sequences) and H5 gs/Gd lineages (4971 sequences) are described. Finally, the molecular composition and frequency of H7 HACS motifs (2819 sequences) are described.

### 5.1 General Characteristics of the H5 and H7 HACS Motif

Haemagglutinin activating proteases with monobasic and polybasic substrate specificity (described above) exhibit a strict substrate requirement at P1. All naturally occurring H5 and H7 AIVs harbour a basic residue at P1, with a strong preference for Arg (Table 3, Table 4, Table 5, Table 6, Table 7). Although Lys is present at P1 in some sequences, the frequency is extremely low, suggesting reduced functional fitness. Specifically, Lys is present at P1 in 0.71% (6/850), 0.02% (1/4971) and 0.43% (12/2819) of H5 American‐lineage, H5 gs/Gd‐lineage, and H7 HACS motifs, respectively. No sequences with Lys at P1 were present in the H5 Eurasian‐lineage dataset analysed. The H5 HACS motif is generally flanked by PQ and GLF at N‐ and C‐termini, respectively. Some variation exists at these flanking positions, which may be indicative of pathotype conversion mechanisms sampling the mutational space prior to expansion of the HACS motif. For the H7 subtype, the HACS motif is generally flanked by PE and GLF at N‐ and C‐termini, respectively. HACS motifs can be categorised into 3 groups depending on the number of basic amino acids it contains [313]: (1) short HACS motif with 1‐2 basic amino acids, (2) mid‐length HACS with 3‐4 basic amino acids, and (3) extended pHACS motif that contain ≥ 5 basic amino acids. LPAIVs typically have short HACS motifs and HPAIVs frequently harbour extended pHACS motifs that generally contain ≥ 5 basic amino acids.

**TABLE 5:** Frequency of American‐lineage H5 HACS motifs and associated neuraminidase subtypes.

	American‐lineage H5 HACS motif^a^	Neuraminidase subtype											HACS motif length	Number of basic amino acids in HACS motif	Frequency	HPAIV^b^
		N1	N2	N3	N4	N5	N6	N7	N8	N9	Mixed	H5				
Extended	P**K**_REKREKR/G		N2										7	5	1	Y
	PQ_RKRKKR/G								N8				6	6	2	Y
	^c^PQ_RKRKTR/G		N2										6	5	3	Y
	^c^PQ_RRKKR/G									N9			5	5	1	Y
Mid‐length	^c^PQ_RKKR/G		N2										5	4	12	Y
	PQ_KKKR/G		N2										4	4	8	Y
	PQ_RRKR/G		N2										4	4	2	Y
	^c^PQ_RKTR/G	N1	N2							N9		H5	4	3	25	N^d^
	P**K**_RKTR/G		N2										4	3	5	N^d^
	PQ_REKR/G		N2	N3									4	3	16	N^d^
	LQ_REKR/G		N2										4	3	1	N^d^
	PQ_KEKR/G		N2										4	3	1	N^d^
	PQ_RRTR/G		N2										4	3	1	U
Short	^c^PQ_RETR/G	N1	N2	N3	N4	N5	N6	N7	N8	N9		H5	4	2	683	N
	^c^P**K**_RETR/G		N2										4	2	1	N
	^c^PQ_KETR/G		N2	N3				N7	N8	N9			4	2	45	N
	^c^PQ_REAR/G	N1	N2					N7	N8	N9			4	2	7	N
	PQ_KETK/G		N2										4	2	6	N
	PQ_RGTR/G		N2					N7	N8				4	2	5	N^e^
	^c^PQ_REIR/G		N2										4	2	2	N
	PQ_REPR/G			N3									4	2	2	N^e^
	^c^PQ_RDTR/G		N2										4	2	1	N
	PQ_KEIR/G		N2										4	2	1	N^e^
	^c^PQ_IETR/G	N1	N2										4	1	18	N^e^
	PQ_GETR/G								N8				4	1	1	N^e^
														**TOTAL**	**850**	

^a^ Basic amino acids flanking the defined HACS motif are in boldface.

^b^ Y = yes. N = no. U = Unknown. Pathotype information not publicly available.

^c^ Sequence also present in H5 Eurasian‐lineage.

^d^ HACS motif likely to be an intermediate between LPAIV and HPAIV.

^e^ Conforms to molecular definition of LPAIV.

**TABLE 6:** Frequency of Eurasian‐Lineage H5 HACS motifs and associated neuraminidase subtypes.

	Eurasian‐lineage H5 HACS motif^a^	Neuraminidase subtype											HACS motif length	Number of basic amino acids in HACS motif	Frequency	HPAIV^b^
		N1	N2	N3	N4	N5	N6	N7	N8	N9	Mixed	H5				
Extended	^c^PQ_REKRRKKR/G		N2										8	7	3	Y
	PQ_RETRRQKR/G			N3									8	5	1	Y
	PQ_RETRRETR/G		N2										8	4	1	N
	PQ_RRRKKR/G	N1	N2										6	6	10	Y
	^d^PQ_RKRKTR/G	N1											6	5	1	Y
	^d^PQ_RRKKR/G		N2										5	5	4	Y
Mid‐length	**H**Q_RRKR/G	N1	N2							N9			4	4	5	Y
	^d^PQ_RKKR/G	N1											5	4	1	Y
	^d^PQ_RKTR/G		N2										4	3	2	N^e^
Short	^d^PQ_RETR/G	N1	N2	N3			N6	N7	N8	N9	Mixed	H5	4	2	266	N
	^d^P**K**_RETR/G											H5	4	2	1	N
	^d^PQ_KETR/G	N1	N2	N3				N7		N9			4	2	18	N
	PQ_RATR/G			N3									4	2	1	N
	^d^PQ_RDTR/G		N2										4	2	1	N
	^d^PQ_REAR/G			N3									4	2	1	N
	PQ_KEAR/G			N3									4	2	1	N
	^d^PQ_REIR/G			N3									4	2	1	N
	^d^PQ_IETR/G		N2										4	1	1	N^f^
														**TOTAL**	**319**	

^a^ Basic amino acids flanking the defined HACS motif in boldface.

^b^ Y = yes. N = no. U = Unknown. Pathotype information not publicly available.

^c^ HACS = motif also present in Gs/Gd/96‐lineage.

^d^ Sequence also present in H5 American lineage.

^e^ HACS motif likely to be an intermediate between LPAIV and HPAIV.

^f^ Conforms to molecular definition of LPAIV.

**TABLE 7:** Frequency of H5 gs/Gd‐lineage HACS motifs and associated neuraminidase subtypes.

Gs/Gd‐lineage H5 HACS motif^a^	Neuraminidase subtype											HACS motif length	Number of basic amino acids in HACS motif	Frequency
	N1	N2	N3	N4	N5	N6	N7	N8	N9	Mixed	H5			
PQ_REREGGRRRKR/G	N1											11	7	7
PQ_REGGRRRKR/G	N1											9	6	2
PQ_GEGRRRKKR/G	N1											9	6	1
PQ_IEGGRRRKR/G	N1											9	5	1
PQ_GEGGRRKKR/G	N1											9	5	1
P**K**_RKRRRKKR/G	N1											8	8	1
PQ_RERRRKKR/G	N1	N2									H5	8	7	418
PL_RERRRKKR/G	N1	N2										8	7	3
PQ_RERRRKKR/T	N1											8	7	1
P**H**_RERRRKKR/G	N1											8	7	1
^b^PQ_REKRRKKR/G	N1											8	7	29
PL_REKRRKKR/G	N1											8	7	1
PQ_RERRRRKR/G	N1											8	7	28
PL_RERRRRKR/G	N1											8	7	4
PQ_RERKRKKR/G	N1											8	7	5
PQ_RERRRKRR/G	N1											8	7	4
PQ_RERRRKRR/**R**	N1											8	7	1
PL_REKRRRKR/G	N1				N5	N6						8	7	4
PQ_GKRRRKKR/G	N1											8	7	4
PQ_RGRRRKKR/G	N1											8	7	2
PQ_RERRRKKK/G	N1											8	7	1
PQ_RERRKKKR/G	N1											8	7	1
PQ_RDRRRKKR/G											H5	8	7	1
PP_RGKRRRKR/G					N5							8	7	1
PQ_EKKRRKKR/G	N1											8	7	1
PQ_RKERRKKR/G	N1											8	7	1
PQ_GERRRKKR/G	N1											8	6	732
P**K**_GERRRKKR/G	N1											8	6	1
P**H**_GERRRKKR/G	N1											8	6	1
P**R**_GERRRKKR/G	N1											8	6	1
LQ_GERRRKKR/G	N1											8	6	1
PQ_GEKRRKKR/G	N1											8	6	261
P**K**_GEKRRKKR/G	N1											8	6	5
**H**Q_GEKRRKKR/G	N1											8	6	4
P**R**_GEKRRKKR/G	N1											8	6	1
PL_GEKRRKKR/G	N1											8	6	1
LQ_GEKRRKKR/G	N1											8	6	1
PQ_RESRRKKR/G	N1											8	6	215
PQ_REERRKKR/G	N1											8	6	114
P**R**_REERRKKR/G	N1											8	6	1
PQ_REGRRKKR/G	N1											8	6	69
PQ_IERRRRKR/G	N1									Mixed		8	6	36
PQ_GERRRRKR/G	N1											8	6	17
PQ_IERRRKKR/G	N1											8	6	15
P**R**_IERRRKKR/G	N1											8	6	2
PQ_REIRRKKR/G	N1											8	6	12
PQ_GKSRRKKR/G	N1											8	6	9
PQ_RESRRKRR/G	N1											8	6	7
PQ_EERRRKKR/G	N1											8	6	6
PQ_GERKRKKR/G	N1											8	6	3
PQ_GERRRKRR/G	N1											8	6	2
PQ_KESRRKKR/G	N1											8	6	2
PQ_RESRRRKR/G	N1											8	6	2
PQ_RVGRRKKR/G	N1											8	6	1
PQ_RERRREKR/G	N1											8	6	1
PQ_RERGRKKR/G	N1											8	6	1
PQ_REEKRKKR/G	N1											8	6	1
PQ_IDRRRRKR/G	N1											8	6	1
PQ_GKNRRKKR/G	N1											8	6	1
PQ_GDRRRKKR/G	N1											8	6	1
PQ_EGRRRKKR/G	N1											8	6	1
PL_RERRRRNR/G								N8				8	6	1
PQ_GEGRRKKR/G	N1											8	5	59
PQ_IEGRRRKR/G	N1	N2										8	5	38
PQ_REGGRRKR/G	N1											8	5	6
PQ_REGGRKKR/G	N1											8	5	2
PQ_EEGRRKKR/G	N1											8	5	2
PQ_REERRKNR/G	N1											8	5	1
PQ_REERRKIR/G	N1											8	5	1
PQ_GERRRIKR/G	N1											8	5	1
PL_IEGRRKKR/G	N1											8	5	1
PL_RKRRRKR/G	N1											7	7	1
PQ_RERRRKR/G	N1	N2				N6			N9	Mixed	H5	7	6	1284
PL_RERRRKR/G	N1	N2	N3		N5	N6		N8			H5	7	6	999
PS_RERRRKR/G						N6						7	6	23
P**K**_RERRRKR/G	N1											7	6	4
PP_RERRRKR/G	N1					N6						7	6	2
LQ_RERRRKR/G	N1											7	6	2
**H**L_RERRRKR/G						N6						7	6	1
SQ_RERRRKR/G	N1											7	6	1
P**R**_RERRRKR/G	N1											7	6	1
L**R**_RERRRKR/G	N1											7	6	1
PQ_RERRRKR/E	N1											7	6	1
PQ_RERRRKR/**R**	N1											7	6	1
PL_REKRRKR/G	N1	N2			N5	N6		N8		Mixed	H5	7	6	328
PQ_REKRRKR/G	N1											7	6	4
PP_REKRRKR/G					N5							7	6	2
PS_REKRRKR/G						N6						7	6	1
PQ_RERRKKR/G	N1											7	6	60
PL_RERRKKR/G						N6			N8			7	6	4
PQ_KERRRKR/G	N1									Mixed		7	6	37
PL_KERRRKR/G	N1					N6						7	6	3
PL_KEKRRKR/G						N6			N8	Mixed		7	6	10
PQ_RERRRRR/G	N1											7	6	5
PL_RERRRRR/G	N1											7	6	2
PL_RGRRRKR/G	N1											7	6	4
PQ_RGRRRKR/G	N1											7	6	2
PQ_REKRKKR/G												7	6	3
PL_REKRRRR/G						N6						7	6	2
PL_RGKRRKR/G		N2										7	6	1
PQ_KERRRRR/G	N1											7	6	1
PQ_GERRRKR/G	N1											7	5	6
PL_GERRRKR/G									N8			7	5	1
PQ_RERRGKR/G	N1											7	5	3
PQ_IERRRKR/G										Mixed		7	5	1
PL_IERRRKR/G		N2										7	5	1
PL_REKGRKR/G						N6						7	5	1
PL_SERRRKR/G		N2										7	5	1
PQ_SERRRKR/G	N1											7	5	1
PL_REGRRKR/G	N1											7	5	1
													**TOTAL**	**4971**

^a^ Basic amino acids flanking the defined HACS motif in boldface.

^b^ Sequence also present in H5 Eurasian‐lineage.

### 5.2 Low Pathogenicity Avian Influenza Virus Short HACS Motifs

Low pathogenicity avian influenza viruses are the predominant pathotype detected, therefore their corresponding sequences constitute 96.6% (821/850), 92.2% (294/319) and 91.0% (2547/2800) of all HACS sequences for American H5, Eurasian H5, and H7 AIVs, respectively. Specifically, H5 LPAIVs with short (mono‐ and di‐basic), and mid‐length (tri‐basic) HACS motifs have been detected, with short di‐basic HACS motifs being the most prevalent (PQ_RXXR/G). The archetypical H5 LPAIV HACS motif, PQ_RETR/G, accounts for 80.4% (683/850) and 83.4% (266/319) of American and Eurasian‐lineage sequence diversity, respectively (Table 5, Table 6), and constitutes a similar frequency of American and Eurasian‐lineage LPAIVs (83.2% (683/821) and 90.5% (266/294), respectively). Variations of this archetypical H5 LPAIV HACS motif typically contain non‐basic amino acid substitutions at P2 and P3.

Similar to H5 LPAIVs, H7 LPAIVs with di‐ and tri‐basic HACS motifs are frequently isolated. Furthermore, one H7 LPAIV HACS motif sequence contains four basic amino acids, though crucially, a non‐basic amino acid is present at the P4 subsite. The consensus H7 LPAIV HACS motif is PE_XPKXR/G and the most numerous H7 LPAIV HACS motif detected is PE_IPKGR/G, representing 43.4% (1224/2819) of all H7 HACS motifs and 48.1% (1224/2547) of H7 LPAIV motifs (Table 4). Although, the H7 LPAIV HACS motif PE_NPKTR/G (all: 26.8% (756/2819), LPAIV only: 29.7% (756/2547)), and to a lesser extent PE_KPKPR/G (all: 6.7% (188/2819), LPAIV only: 7.4% (188/2547)) are also frequently detected. All LPAIVs that harbour short HACS motifs generally replicate in the mucosal epithelia and are of low pathogenicity in gallinaceous birds.

### 5.3 Mid‐Length HACS Motifs Are Likely Transitional in Naturally Occurring Avian Influenza Viruses

A small proportion of mid‐length HACS motifs (3‐4 basic residues) are present in naturally occurring H5 and H7 AIVs. Mid‐length HACS motifs constitute 8.4% (71/850), 2.5% (8/319) and 10.6% (298/2819) of H5 American‐lineage, H5 Eurasian‐lineage and H7 HACS motifs, respectively. The genesis of mid‐length HACS motif arising from basic amino acid substitutions in the consensus H5 LPAIV HACS motif PQ_RXXR/G at subsite(s) P2 or P3, or in the consensus H7 LPAIV HACS motif PE_XPKXR/G at subsite P2 and potentially P4 of the HACS may lead to initial, albeit inefficient, interactions with enzymes with polybasic specificity prior to evolving into highly pathogenic forms. Mid‐length HACS motifs have been detected in both LPAIVs and HPAIVs, suggesting that mid‐length HACS motifs are likely to be transitional in naturally occurring populations [315].

The most prevalent mid‐length pHACS motif detected in American‐lineage H5 AIVs is PQ_RKKR/G (HPAIV: 43.3% 12/19 sequences; all: 12/850 sequences) and was detected during the 2004 and 2012‐13 HPAIV epornitics. The HACS motif detected during the 2004 H5 epornitic in the USA was the mid‐length motif, PQ_RKKR/G (Table 1). Phylogenetic analysis revealed that the closest known virus was of low pathogenicity and had a tri‐basic mid‐length HACS motif, PQ_REKR/G. Although the HPAIV from this epornitic did not cause severe disease in chickens, it was molecularly classified as highly pathogenic [48]. HPAIVs with similar mid‐length HACS motifs isolated during the 1959 (PQ_RKKR/G), 1983–84 (PQ_KKKR/G), 2012 (PQ_RRKR/G) and 2015–2016 (HQ_RRKR/G) epornitics (Table 1) do cause severe disease in chickens and turkeys. This supports that the stepwise accumulation of substitutions in the HACS motif (e.g.,: PQ_REXR/G → PQ_REKR/G → PQ_RKKR/G) readily occurs in naturally occurring populations [276] and that the genesis of viruses with mid‐length HACS motif exhibit pathogenic potential [276, 285, 288]. Experimentally, H5 and H7 AIVs with mid‐length motifs seem to be unstable and are rapidly outcompeted by viruses that harbour extended HACS motifs (≥ 5 basic amino acids) [285, 316]. Using reverse genetics, an H7 virus that contained a mid‐length HACS motif (PE_IPKKR/G) that was serially passaged in embryonating chicken eggs ultimately yielded HPAIV HACS motif variants containing multiple basic amino acid insertions (PE_IPKKKKR/G and PE_IPKRKKR/G) [316]. Emergence of HPAIVs from LPAIVs precursors has been associated with this rare mid‐length motif ‐ it was also detected in a potential LPAIV precursor that was cocirculating during the 1976 HPAIV epornitic in Australia [287]. Similarly, in vivo passage of H5 AIVs with mid‐length motifs in chickens lead to rapid replacement of mid‐length motifs by virus populations with extended motifs (EG: PQ_RKTR/G → PQ_RKRKTR/G; PQ_RKKR/G → PQ_RRKKR/G) [285]. Interestingly, an H9N2 LPAIV (A/chicken/Israel/810/2001) that contains a mid‐length pHACS motif, RSKR/G, was demonstrated to be cleaved by furin in vitro [317]. Additional reverse genetics studies with mid‐length HACS motifs on various genetic backgrounds/constellations (LPAIV and HPAIV) may help to further clarify conditions that support the emergence and selection of extended motifs in viral populations. Furthermore, biochemical studies characterising monobasic and polybasic enzymatic activity (turnover rate) for mid‐length motifs would likely provide insight into the mechanisms contributing to pathotype conversion events.

### 5.4 High Pathogenicity Avian Influenza Virus Extended pHACS Motifs

The pHACS motif of HPAIVs typically contains five or more basic consecutive amino acids although some H5 Eurasian‐lineage and H5 American‐lineage (PQ_RKKR/G, PQ_KKKR/G, PQ_RRKR/G) and H7 HPAIVs (PE_IPKRRR/G) with mid‐length motifs are described. There is relatively low sequence diversity of extended pHACS in American‐lineage HPAIVs. Four extended pHACS motifs constitute 24.1% (7/29) American‐lineage HPAIV motifs. The most numerous extended pHACS motif is PQ_RKRKTR/G (3/7) and was detected during the 1994‐1995 Mexico epornitic. Of the Eurasian‐lineage H5 HPAIVs with extended pHACS, the most prevalent motif is PQ_RRRKKR/G (10/26 HPAIV, 38.5%; 10/319 sequences) and was detected during the 1997–98 Italian epornitic.

**FIGURE 3:**
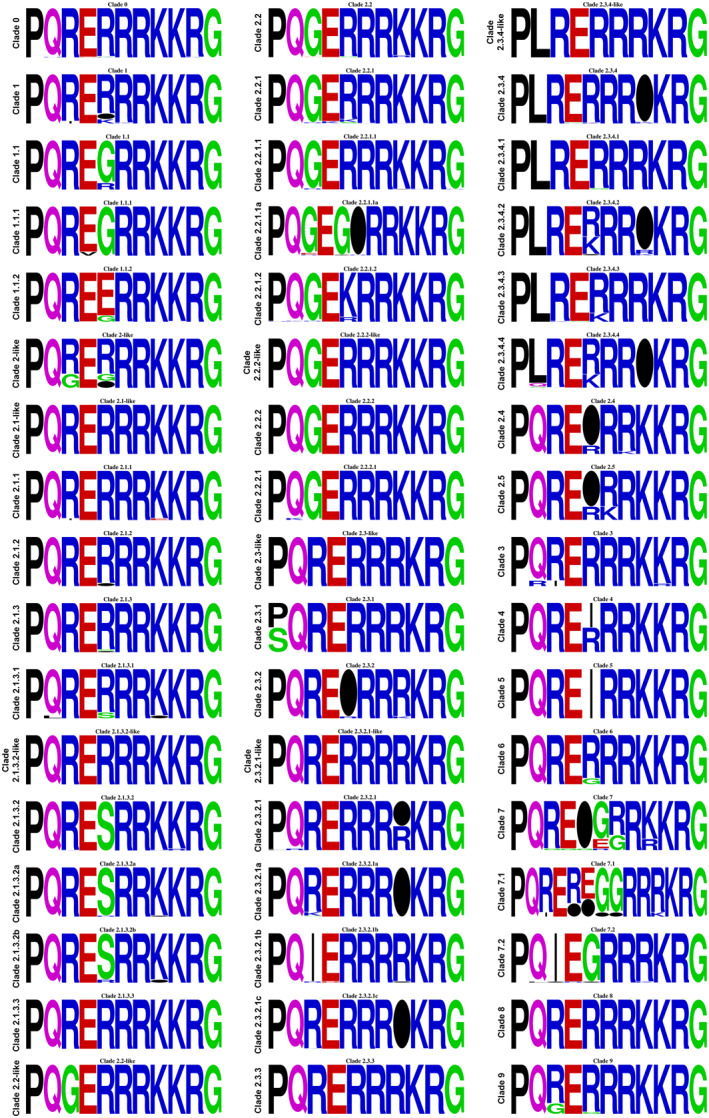
Sequence logo representation of clade‐specific amino acid variation in the haemagglutinin cleavage site motif of naturally occurring gs/Gd‐lineage H5 HPAIVs. Protein sequences were downloaded from Influenza Research Database [314] and sequences with ambiguous base calls, laboratory‐generated, and duplicate sequences were omitted (*n* = 4971). Sequence logos were generated using WebLogo [318]. Black circles indicate amino acid deletion.

The molecular composition of the gs/Gd‐lineage HPAIV pHACS motif was dominated by the PQ_RERRRKKR/G motif for 20+ years. This archetypical H5 gs/Gd‐lineage pHACS motif which is found in numerous clades and subclades contains 8 amino acids, seven of which are basic amino acids (Figure 3, Table S1). Whilst the PQ_RERRRKKR/G motif was initially the most prevalent in naturally occurring gs/Gd‐lineage H5 HPAIVs (8.4%, 418/4971 sequences), clade‐dependant pHACS variants have emerged (Figure 3, Table S1). The dominant pHACS motif detected in clade 2.2 and derivatives thereof (clades 2.2‐like, 2.2.1, 2.2.1.1, 2.2.1.1a, 2.2.1.2, 2.2.2, 2.2.2‐like, 2.2.2.1) contain a substitution from Arg to Gly at P8, PQ_GERRRKKR/G (14.7%, 732/4971) and PQ_GEKRRKKR/G (5.3%, 261/4971) (Figure 3, Table S1). Variants of these two motifs contain basic amino substitutions at P9 or P10, suggesting that mechanisms involved in virus evolution were sampling the mutational space. A substitution at P6 from Arg to Gly to form the pHACS motif PQ_REGRRKKR/G was initially detected in clade 0 and became the dominant pHACS in clades 1.1, 1.1.1 and 7. The P6 substitution Arg to serine present in clades 2.1.3, 2.1.3.1, 2.1.3.2, 2.1.3.2a‐b (4.3%, 215/4971) (Figure 3, Table S1), and became the dominant motif in clades 2.1.3.2 and 2.1.3.2a‐b. Experimentally, serine at subsite P6 increased HA cleavage efficiency compared to Gly and even Arg [319]. Finally, isoleucine (clades 4 and 5) and glutamic acid (Glu) (clade 1.1.2) are the dominant amino acids present at subsite P6 that have become fixed in the population. Polymorphism at subsite P6 suggests plasticity at this subsite, and non‐basic amino acids are tolerated.

The plasticity of P6 subsite may have facilitated the emergence and fixation of the atypical pHACS motif now (as of March 2023) dominant in contemporary H5 HPAIV clades. The composition of the gs/Gd‐lineage HPAIV pHACS motif was predominantly PQ_RERRRKKR/G for 20+ years, although recently, the pHACS motif PL_RERRRKR/G (20.1%, 999/4971), initially detected in clade 2.1.3.1, has become fixed in currently circulating populations, indicating that gs/Gd‐lineage HPAIVs harbouring this pHACS motif may have a fitness advantage in their ecological niche. This atypical pHACS motif contains seven amino acids, six of which are basic amino acids. Interestingly, the acidic amino acid, Glu, is present at the polymorphic P6 subsite. The leucine (Leu) in the contemporary atypical pHACS motif may be a compensatory substitution to compensate for a suboptimal acidic residue (Glu) present at P6. A variant of this motif, PQ_RERRRKR/G (25.9%, 1284/4971) was initially detected in a clade 12,005 Vietnamese H5N1 isolate. Subsequently, this motif was frequently detected in clades 2.1.3.2a, 2.1.3.2b, 2.2.1.1, 2.3‐like, 2.3.1, 2.3.2, 2.3.2.1, 2.3.2.1‐like, 2.3.2.1a, 2.3.2.1b, 2.3.2.1c and 2.3.4.4. Another similar pHACS motif, PQ_RERRKKR/G was also detected in clade 1 H5N1 HPAIVs in Vietnam in 2005. The genesis of these pHACS motif could have arisen from insertions into the LPAIV HACS leading to expansion of the HACS motif, or alternatively, it may have been generated from a deletion of a P2 or P3 Lys residue from the archetypical motif, PQ_RERRRKKR/G. The omission of a Lys residue seems to precede the appearance of Leu at P8 and the capacity of gs/Gd‐lineage HPAIVs to reassort with multiple NA subtypes, suggesting that altered pHACS cleavage dynamics may have contributed to the emergence of H5Nx HPAIVs.

Similar to H5 HPAIVs, extended H7 HPAIV pHACS motifs also contain five or more basic amino acids (Table 4) (8.8%, 252/2819) and cluster into two subgroups based on the likely molecular event driving expansion of the HACS motif. Subgroup I is dominated by extended H7 pHACS arising from recombination events with viral or host nucleic acids and subgroup II is dominated by extended H7 pHACS likely formed by polymerase slippage and insertion events. Extended H7 pHACS motifs clustering in subgroup I are 11–15 amino acids in length (which is notably longer than pHACS motifs generated by polymerase slippage events), contain 5 or more basic amino acids, and are generally associated with the N3 subtype (discussed below) (32.5%, 82/252) (Table 4). Furthermore, basic amino acids are generally present at key subsites rather than having consecutive basic residues. pHACS motifs in subgroup I are populated primarily by North American isolates detected during the 2002 Chilean [43], 2004 and 2007 Canadian [47, 52], 2012 (ongoing) Mexican [58], and 2017 and 2020 US [66, 71] HPAIV outbreaks. Of these, the Mexican H7 HPAIV outbreak has been the largest in terms of duration and geographical spread. Consequently, the pHACS motif, PE_NPKDRKSRHRRTR/G, and variations thereof detected during this outbreak are the most prevalent in subgroup I (34.2%, 28/82). Although, sampling intensity may influence this result.

Extended H7 pHACS motifs clustering in subgroup II are 7‐9 amino acids in length, contain 5 or more consecutive basic amino acids, and are associated with numerous NA subtypes (67.5%, 170/252) (Table 4). The ancestral, Australian, European, and Asian H7 HPAIVs cluster in subgroup II H7 extended pHACS motifs. The most numerous pHACS motif in subgroup II is PE_VPKRKRTAR/G (33.5%, 57/170), which emerged during the fifth wave of the Chinese H7N9 outbreak. However, as discussed above for H7 extended pHACS motifs detected during the Mexico H7 HPAIV outbreak, the sampling intensity that occurred during the H7N9 outbreak also likely skews the frequency and hence dominant pHACS in subgroup II. The ancestral H7 pHACS motifs are 7‐8 amino acids long, contain 5‐6 basic amino acids, and are predominately associated with the N7 subtype. The H7 ancestral pHACS motifs include FPV/Brescia/1902, FPV/Weybridge/1934, and FPV/Dutch/1934 (PE_IPKRKKR/G and PE_LPKKRRKR/G), FPV/Rostock/1934 (PE_PSKKRKKR/G), England/1963 (PE_TPKRRRR/G), and those detected during the 1979 German HPAIV outbreak (PE_IPKKKKKKR/G, PE_TPKKKKKKR/G, PE_IPKKRKKR/G, PE_IPKKKKR/G, PE_IPKRKKR/G). Notably, pHACS with various numbers of basic amino acids were detected during the 1979 German HPAIV epornitic, suggestive of evolutionary mechanisms actively sampling the mutational space. Only one H7 pHACS motif of North American origin (2016 Indiana USA HPAIV outbreak) is represented in subgroup II (PE_NPKKRKTR/G). Australian H7 HPAIVs subgroup II detected during the 1970s and 1980s harbour PE_IPKKREKR/G pHACS, whereas Australian H7 HPAIVs detected from 1992 onwards contain one less amino acid in the pHACS (PE_IPKKKKR/G and PE_IPRKRKR/G). The omission of an acidic residue in the pHACS motif likely optimised substrate binding dynamics with activating enzymes, leading to increased viral fitness. Biochemical analysis of binding dynamics and enzymatic turnover rates of H5 and H7 polybasic substrates would shed further light on mechanisms leading to fixation of a particular pHACS motif in HPAIV populations.

### 5.5 NA Subtype and Associated HACS Motif Sequences

**FIGURE 4:**
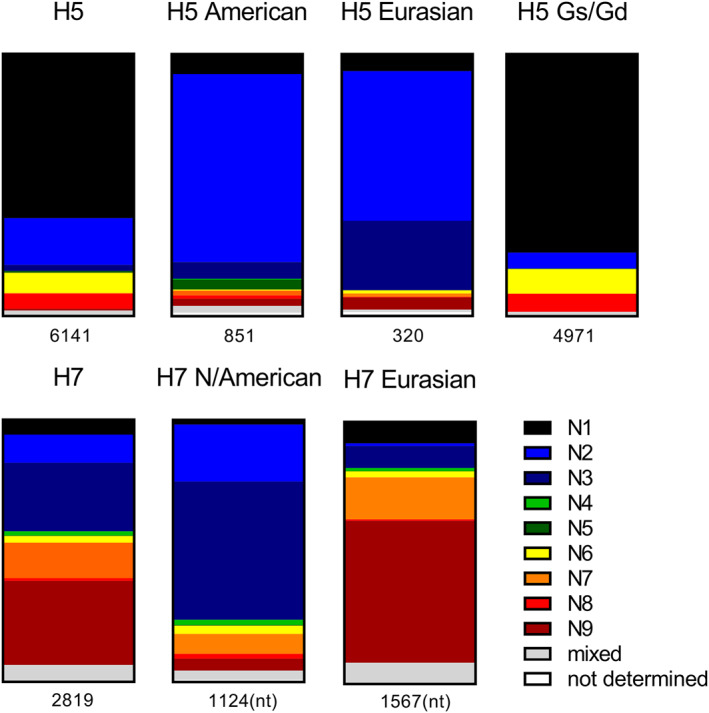
Neuraminidase subtypes associated with major H5 and H7 AIV lineages. HA protein sequences were downloaded from Influenza Research Database [314] and sequences with ambiguous base calls, laboratory‐generated, and duplicate sequences were omitted (*n* indicated below subtype/lineage). To examine NA subtypes associated with major H7 lineages, H7 HA nucleotide (nt) sequences were downloaded from Influenza Research Database [314], filtered as above, and phylogenetic tree was generated using RaxML with 100 bootstrapping replicates. North American and Eurasian lineages were identified and associated NA subtype examined.

The functional balance of HA and NA activity influences viral fitness, and changes in the functional activity of HA is typically followed by changes in the enzymatic activity of NA (and conversely) to maintain viral fitness [320, 321, 322, 323]. The most frequently associated NA subtype with H5 and H7 AIV HA sequences is N1 (62.8%, 3853/6140) and N9 (32.2%, 906/2819), respectively, although the predominant NA subtype changes depending on major sub‐lineage (Figure 4). Specifically, North American H7 and Eurasian H7 lineages are predominately associated with N3 (52.8%, 593/1124) and N9 (54.2%, 849/1567) subtypes, respectively (Figure 4). Interestingly, there seems to be an association between H7 HPAIVs generated by recombination events and the N3 subtype (Table 4).

In poultry and monitored wild bird populations, American H5, Eurasian H5 and gs/Gd‐lineages have been predominantly associated with N2 (71.8%, 610/850), N2 (57.1%, 182/319) and N1 (75.8%, 3767/4971) neuraminidase subtypes, respectively (Figure 4). The prototypical gs/Gd‐lineage pHACS motif, PQ_RERRRKKR/G, was most frequently associated with the N1 subtype for many years, however, contemporary gs/Gd‐lineage HPAIVs (e.g., clade 2.3.4.4) are now detected with numerous NA subtypes, including N1‐3, N5‐6 and N8‐9 (Table S1). Contemporaneously, the atypical pHACS motif PL_ RERRRKR/G that has a Lys deletion in addition to a glutamine to Leu substitution at P8 which flanks the pHACS motif [324] (plus an additional 10 pHACS motif variants with substitutions at P8 or P9, Table 7, Table S1) became fixed in the contemporary 2.3.4.4b clade. This pHACS motif may lead to altered cleavage activation and in turn have influenced HA NA balance, facilitating functional fitness with a wider array of NA subtypes. In a similar manner, the prototypical LPAIV HACS motifs (H5 ‐ PQ_RETR/G; H7—PE_IPKGR/G, Table 3, Table 4, Table 5, Table 6), are frequently detected with numerous NA subtypes. Biochemical and functional studies examining HA and NA balance (such as HA activation, receptor binding, NA activity) may inform why these HACS motifs are frequently detected with numerous subtypes. An infection study with recombinant H5 clade 2.3.4.4 HPAIV with various NA subtypes suggested that reassortants harbouring N6 and N8 subtypes exhibited increased virulence (survival time decreased from 72 (H5N1) or 96 h (H5N2) to 48 h) and transmitted more efficiently to contact birds [325]. However, this result is likely to be highly strain dependent as chickens routinely succumb to gs/Gd H5N1 viruses within 24–48 h and other genes likely contribute to virulence [35, 285, 326, 327, 328]. Another study suggested that H5N6 HPAIVs acquired dual receptor specificity and transmitted efficiently to contact ferrets [329]. Alternatively, frequent reassortment may be due to the gs/Gd‐lineage becoming endemic in wild birds, therefore it has a chance to mix with various AIV lineages that are not present in poultry. Individual combinations may result in better fitness, but the process may be stochastic.

## 6 Conclusions

H5 and H7 AIVs continue to evolve molecularly, leading to the emergence and selection of isolates with novel characteristics. Coupled with the change in HPAIV ecology from predominately emerging in gallinaceous poultry populations, to being maintained in wild birds, the threat of HPAIV to humans and animals is increasing. This threat is exemplified by numerous avian and mammalian species succumbing to H5 clade 2.3.4.4b HPAIV infection during the current outbreak in the USA [330, 331] and now spillover to livestock including dairy cows [332] and a backyard pig [333]. At present, AIVs isolated from wild bird species that carry AIV, and the infection dynamics of the virus in wild bird species remains understudied—expanding research in avian species beyond that of poultry is crucial to our understanding of infection dynamics and emergence of novel isolates. Furthermore whilst antigenicity is closely monitored to ensure efficacy of current vaccines, basic research characterising other phenotypic attributes using classical and computational sciences such as infectious disease modelling and machine learning will strengthen our understanding of HPAIV emergence. Functional characterisation of HA structure and cleavage, receptor specificity, NA activity, replicative ability, environmental stability, and immune response to infection will complement antigenic characterisations and provide additional insights into the attributes that confer fitness and selection of HPAIVs in various avian hosts and environments (habitats). Increased research to understand the evolution and emergence of novel strains is crucial to understanding risk and pandemic potential. An in‐depth understanding of the molecular virology of AIVs is necessary to prepare for and counter the global threat of H5 and H7 AIVs.

## Author Contributions

Conceptualization, J.M.L.; Formal analysis, J.M.L.; Writing–Original Draft, J.M.L.; Writing–Review & Editing, J.M.L. and E.S.; Visualization, J.M.L.

## Conflicts of Interest

The authors declare no conflicts of interest.

## Supporting information

Table S1

## Data Availability

The data that support the findings of this study are available from the corresponding author upon reasonable request. GISAID data that support the findings of this study are available from https://gisaid.org/. Restrictions apply to the availability of these data, which were used under license for this study.
