# Supplementary figures and images for: Molecular Evolution of the H5 and H7 Highly Pathogenic Avian Influenza Virus Haemagglutinin Cleavage Site Motif
Source: Rev Med Virol. 2024 Dec 27;35(1):e70012. doi: 10.1002/rmv.70012 (PMC11680514; doi:10.1002/rmv.70012)

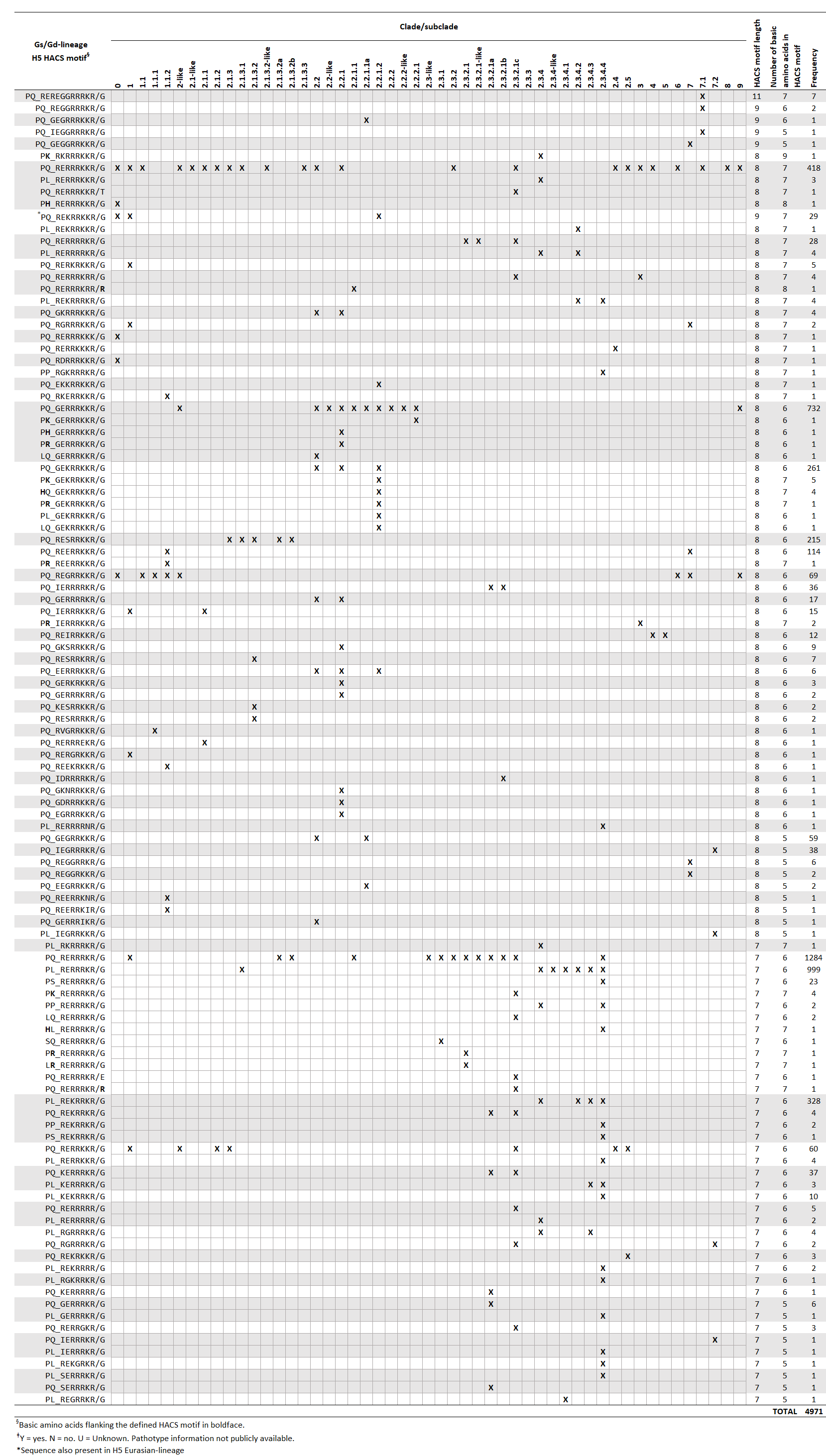

Supplement: Supplementary file 1 — Table S1 [file RMV-35-e70012-s001.tif]
